# Structural Characterization of Two Metastable ATP-Bound States of P-Glycoprotein

**DOI:** 10.1371/journal.pone.0091916

**Published:** 2014-03-14

**Authors:** Megan L. O’Mara, Alan E. Mark

**Affiliations:** 1 School of Chemistry and Molecular Biosciences (SCMB), The Institute for Molecular Biosciences (IMB), The University of Queensland, Brisbane, Queensland, Australia; 2 School of Mathematics and Physics (SMP), The Institute for Molecular Biosciences (IMB), The University of Queensland, Brisbane, Queensland, Australia; 3 The Institute for Molecular Biosciences (IMB), The University of Queensland, Brisbane, Queensland, Australia; University of Technology Sydney, Australia

## Abstract

ATP Binding Cassette (ABC) transporters couple the binding and hydrolysis of ATP to the transport of substrate molecules across the membrane. The mechanism by which ATP binding and/or hydrolysis drives the conformational changes associated with substrate transport has not yet been characterized fully. Here, changes in the conformation of the ABC export protein P-glycoprotein on ATP binding are examined in a series of molecular dynamics simulations. When one molecule of ATP is placed at the ATP binding site associated with each of the two nucleotide binding domains (NBDs), the membrane-embedded P-glycoprotein crystal structure adopts two distinct metastable conformations. In one, each ATP molecule interacts primarily with the Walker A motif of the corresponding NBD. In the other, the ATP molecules interacts with both Walker A motif of one NBD and the Signature motif of the opposite NBD inducing the partial dimerization of the NBDs. This interaction is more extensive in one of the two ATP binding site, leading to an asymmetric structure. The overall conformation of the transmembrane domains is not altered in either of these metastable states, indicating that the conformational changes associated with ATP binding observed in the simulations in the absence of substrate do not lead to the outward-facing conformation and thus would be insufficient in themselves to drive transport. Nevertheless, the metastable intermediate ATP-bound conformations observed are compatible with a wide range of experimental cross-linking data demonstrating the simulations do capture physiologically important conformations. Analysis of the interaction between ATP and its cofactor Mg^2+^ with each NBD indicates that the coordination of ATP and Mg^2+^ differs between the two NBDs. The role structural asymmetry may play in ATP binding and hydrolysis is discussed. Furthermore, we demonstrate that our results are not heavily influenced by the crystal structure chosen for initiation of the simulations.

## Introduction

P-glycoprotein (P-gp) is an ATP Binding Cassette (ABC) multidrug exporter that uses the energy derived from ATP binding and hydrolysis to power the efflux of substrates across the cell membrane. Since 2006, structural models of P-gp and several homologous ABC exporters have been elucidated using X-ray diffraction (XRD) techniques using crystals obtained from protein solubilized in detergents [1]–[5]. This set of XRD structural models has been solved under a range of experimental conditions, including in the presence and absence of ATP analogues. Collectively they demonstrate the conserved ABC exporter architecture, in which the two TMDs and two NBDs are connected via a domain swapping topology, and the inherent flexibility of this class of proteins. The structures currently available can be classified according to whether they are nucleotide-free or nucleotide bound, and whether the transmembrane pore is accessible from either the inside of the membrane (inward-facing) or outside of the membrane (outward-facing). The inward-facing conformation has been observed in both the presence and absence of nucleotide, whereas all outward-facing conformations are nucleotide bound [2]–[5]. XRD structural models of the inward-facing nucleotide free conformation of P-gp also show large separations between the NBDs [2], [6]–[8]. However, in MD simulations of nucleotide free P-gp in a membrane environment, O’Mara and Mark found that the NBDs spontaneously form a contact interface. This NBD interface is similar to that of the nucleotide bound inward-facing conformations of ABCB10 and TM287/288. Their results suggest that the large separation of the P-gp NBDs may be an artifact of the crystallization conditions [9]. Based on this, it was suggested that the physiological conformation of P-gp most likely resembles that observed in the nucleotide bound XRD structures of ABCB10 and TM287/288. In these two inward-facing nucleotide bound structures, the nucleotide is bound solely to the Walker A motif of one NBD and the two NBDs are in close proximity to each other [3], [4]. In the outward-facing nucleotide bound XRD conformations of P-gp homologues, the transmembrane pore is accessible only from the extracellular environment. The two NBDs form a tight interface with two molecules of an ATP analogue sandwiched between them, forming direct contacts with the Walker A motif of one NBD and the Signature motif of the opposite NBD [1], [5], [10]. This high-affinity “nucleotide sandwich dimer” conformation was first predicted by MD simulations [11] and later confirmed by the XRD structures of the Rad50 and MJ0796 NBDs [12], [13]._ENREF_10 Together these inward- and outward-facing XRD structural models may provide static snapshots of distinct points in the transport cycle, but provide only limited insight into the structural transitions that lead to transport itself. In addition, it has been questioned whether these XRD structural models do in fact represent physiologically relevant conformations [9], [14].

It is well established that P-gp and other ABC exporters most likely exist in an ATP-bound conformation under physiological conditions [14], [15]. However, little mechanistic data is available related to either the dynamics of substrate transport or the nucleotide catalytic cycle itself. This has lead to the propagation of two conflicting mechanistic models for nucleotide binding and hydrolysis, neither of which can be excluded based on the available biochemical, biophysical or structural data [16], [17]. For example, current studies are unable to distinguish whether the transport rates of P-gp and other ABC exporters are limited by NBD dimerization or by ATP hydrolysis. Investigations of the association kinetics of isolated MJ0796 NBDs demonstrated a dynamic equilibrium between the monomeric and dimeric states [17], but the dimerization kinetics of the full-length ABC transporters – both importers or exporters – are not known. Vanadate trapping studies of the nucleotide have demonstrated that both ATP binding sites are catalytically active [18]. Other studies have attempted to biochemically characterize a range of proposed intermediate states, including an asymmetric occluded state [19]. While these have significantly increased the breadth of experimental data available they have not answered the critical question of how ATP binding and hydrolysis is coupled to the structural changes that drive drug transport, or the structural transitions these transporters undergo on ATP binding, hydrolysis, or the subsequent release of ADP and Pi.

A key feature of P-glycoprotein (P-gp) is its low basal rate of ATP hydrolysis, which is stimulated by up to 10 fold by the presence of a transport substrate. Purified P-gp reconstituted into proteoliposomes containing a mixture of either egg phosphatidylcholine and cholesterol, or DPPC and cholesterol, has a V_max_ of 76 +/–10 and 104 +/– 37 nmol⋅min^−1^⋅mg^−1^, respectively [20]. When considering a single molecule of P-gp, this gives average rates of 0.22 +/– 0.03 and 0.29 +/– 0.09 ATP molecules hydrolyzed per molecule of P-gp per second (ATP⋅P-gp^−1^⋅s^−1^). Based on these hydrolysis rates it is clear that long-timescale MD simulations, which typically sample nanosecond to microsecond timescales, cannot be used to model the full transport cycle. They can however be used to examine in detail specific states during the transport cycle and how these are perturbed by the binding of ATP. Here we address the question of what is the dominant ATP-bound conformation of P-gp in the absence of transport substrates. This information is vital to determine the physiological conformation of P-gp during drug uptake and binding.

Specifically, in this study we have examined the effect of Mg^2+^ and ATP binding on the structure of murine based on the P-gp XRD structural model (PDBid 3G5U, chain A) [2] using nonbiased molecular dynamics (MD) simulation techniques. In multiple simulations we observed the formation of two alternative metastable conformations of P-gp. The first conformation closely resembles the XRD structural models of ABCB10 and TM287/288 in which the transporter adopts a nucleotide bound inward-facing conformation. In the second, the binding of ATP is associated with an asymmetric NBD dimerization, where ATP is bound between the Walker A motif of NBD2 and the Signature motif of NBD1 (Binding Site 2 or BS-2), but the NBDs do not interact at the other ATP binding site (Binding Site 1 or BS-1). We characterize these two P-gp conformations in relation to the available structural and biochemical data. We show that the simulation results are not heavily influenced by the choice of the P-gp XRD structure used to initiate the simulation; and that they satisfy the majority of the available biochemical data. Differences in ATP binding and Mg^2+^ coordination between the two homologous NBDs are examined in terms of asymmetries in the initial structures and the evolution of the conformations with time. In all simulations examined, the spontaneous interaction between the Walker A-bound ATP and the opposite Signature motif occurs at Binding Site 2, but not at Binding Site 1. Based on these simulations, we propose that the rate-limiting step in the formation of an ATP sandwich dimer may be the binding of the NBD2 Signature motif with the second ATP molecule, which is bound at the NBD1 Walker A motif.

## Materials and Methods

### Simulation details

All molecular dynamics (MD) simulations were performed using the GROMACS (Groningen Machine for Chemical Simulation) package, version 3.3.3 [21], using the GROMOS 54A7 force field [22] for peptides. The simple point charge (SPC) water model [23] was used to describe the solvent water. All simulations were performed under periodic boundary conditions in a rectangular box. The dimensions of the box were chosen such that the minimum distance to the box wall was at least 1.0 nm. A twin-range method was used to evaluate the non-bonded interactions. Interactions within the short-range cutoff of 0.9 nm were updated every step. Interactions within the long-range cutoff of 1.4 nm were updated every 3 steps together with the pair list. A reaction field correction was applied using a relative dielectric constant of *ε_r_*  =  78.5 to minimize the effect of truncating the electrostatic interactions beyond the 1.4 nm long range cutoff [24]. The SHAKE algorithm [25] was used to constrain the lengths of the covalent bonds. The geometry of the water molecules was constrained using the SETTLE algorithm [26]. In order to extend the timescale that could be simulated, explicit hydrogen atoms in the protein were replaced with dummy atoms, the positions of which were calculated each step based on the positions of the heavy atoms to which they were attached. This eliminates high frequency degrees of freedom associated with the bond angle vibrations involving hydrogens, allowing a time step of 4 fs to be used to integrate the equations of motion without affecting thermodynamic properties of the system significantly [27]. The simulations were carried out in the NPT-ensemble at T  =  300 K, and P  =  1 bar. The temperature and pressure were maintained close to the reference values by weakly coupling the system to an external temperature and pressure bath using a relaxation time constant of 0.1 ps [28] and 0.5 ps, respectively. The pressure coupling was semi-isotropic. Data was collected every 40 ps for analysis. Images were produced using VMD [29].

The protonation states of all ionizable residues were assigned as described previously [9]. Specifically, at neutral pH three histidine residues, His 149 and the catalytic histidines His583 and His1228, were doubly protonated. Residues 1 to 32, 1273 to 1284, and 58 residues of the subunit linker were not observed crystallographically. The termini were not modeled, as there is no information on the structure of these regions. These regions also contained the signal anchor and the His-tag sequences. The subunit linker was also omitted from the simulations. This was because it has been shown experimentally that P-gp is fully functional in the absence of the linker [30]–[33]. In addition, the ability to model loops accurately is poor for loops containing more than 8 to 12 amino acids [34], [35]. Including the linker would, therefore, risk biasing the simulations unnecessarily. Nevertheless, the termini of the peptide chains were neutralized by artificially protonating the C-termini and deprotonating the N-termini to avoid the introduction of inappropriate charges within the protein, as described previously by O’Mara and Mark [9].

The P-gp crystal structure (PDBid 3G5U-a) was inserted into a lipid bilayer containing a 10:1 ratio of POPC (2-oleoyl-1-palmitoyl-*sn*-glycero-3-phosphocholine) [36] and cholesterol [9]. One molecule of ATP was placed in close proximity to the Walker A motifs of each NBD, in the orientation given by the MalK crystal structure [37]. Mg^2+^ was placed in the vicinity of each of the four Mg^2+^ binding sites in the NBDs determined from previous MD simulations [9]. The system was solvated with SPC water and sufficient Cl^−^ counterions added to ensure the overall charge neutrality of the system. Further ions were added to adjust the concentration of the solution to 150 mM NaCl and 1.5 mM MgCl_2_.

The system was equilibrated as follows. Initially 500 steps of steepest descent energy minimization were performed to relax the solvent with all the heavy atoms within P-gp restrained using a harmonic potential with a force constant of 1000 kJ·mol^−1^·nm^−2^. Then a series of 1 ns simulations were performed in which the force constant was progressively reduced (1000, 100, 50 kJ·mol^−1^·nm^−2^). To allow ATP to fully associate with the Walker A and B motifs, each system was simulated for a further 7 ns in which the backbone atoms of P-gp were restrained using a harmonic potential with a force constant of 50 kJ·mol^−1^·nm^−2^. The restraints were then removed and new velocities were assigned. Initially, four independent MD simulations (Runs A to D) were performed each for a period of 90 ns.

In the first 30 ns of Runs C and D1, the ATP molecule bound to the NBD2 Walker A motif and to the NBD1 Signature motif, inducing closure of the NBD2 ATP binding site. This conformation was maintained throughout the remainder of the simulation (90 ns). To examine this state in more detail, Run D1 was branched after 30 ns into three separate simulations. New velocities were assigned and each system was simulated for a further 90 ns (Runs D2 to D4). The original Runs C and D1 were also extended for an additional 30 ns so each system was simulated for a total of 120 ns. Table 1 gives a summary of all simulations performed, together with the sequence motifs binding ATP at each of the ATP binding site at the end of each simulation.

**Table 1:** The starting conformation of P-gp and the motifs in direct contact with ATP at each ATP binding site at the end of the simulation period.

Simulation	Initial conformation	Start (ns)	End (ns)	Binding Site 1 (distance nm)	Binding Site 2 (distance nm)
	3G5U-a			3.9	3.9
Run A	3G5U-a + ATP	0	90	WA (4.0)	WA (3.0)
Run B	3G5U-a + ATP	0	90	WA (3.4)	WA (3.6)
Run C	3G5U-a + ATP	0	120	WA & S (2.0)	WA & S (1.8)
Run D1	3G5U-a + ATP	0	120	WA (3.1)	WA & S (2.1)
Run D2	Run D, 30 ns	30	120	WA (3.0)	WA & S (2.1)
Run D3	Run D, 30 ns	30	120	WA (3.0)	WA & S (2.1)
Run D4	Run D, 30 ns	30	120	WA (2.9)	WA & S (2.5)

***NOTE:*** WA: Walker A motif; S: Signature motif.

Binding Site 1 is the NBD1 Walker A (WA) and NBD2 Signature (S) motif.

Binding Site 2 is the NBD2 Walker A and NBD1 Signature motif.

The distance given is the distance between the center of mass of the Walker A and Signature motif at the end of the simulation.

### Analysis

#### Root mean square deviation (RMSD)

To compare the relative difference between conformations in the trajectories or clusters of conformations extracted from the simulations, the RMSD was calculated using the method of Maiorov and Crippin [38] after first fitting each conformation from the trajectory to a reference structure or domain.

#### Cluster Analysis

To determine the relative populations of specific conformations sampled, the trajectories were clustered using the method of Daura *et al.* [39], [40]. Two conformations were considered neighbors if the backbone RMSD between the two conformations was <0.4 nm, in line with previous studies on this system [9]. To eliminate the possibility of the clusters being biased by the fact all simulations were initiated from the same configuration, the first 10 ns of each trajectory was discarded. The coordinates of the membrane-embedded central structure of the most populated cluster from the trajectories of Runs A, B, C and D is available for download from the Automated Topology Builder and Repository website [41].

#### Solvent Accessible Surface Area Analysis

The average solvent accessible surface area of membrane-embedded P-gp was calculated using the method of Shrake and Rupley [42] with a probe of radius 0.14 nm. The contribution per atom to the solvent accessible surface of the overall system (P-gp, POPC and cholesterol) was determined and the total surface area estimated from the sum of the individual atomic contributions. The initial solvent accessible surface area was taken to be the solvent accessible surface area of membrane-embedded P-gp in the crystallographic conformation.

#### Separation of the NBDs

The distance between the ATP binding sites in the NBDs was measured as the distance between the center of mass of the Walker B motif from one NBD and the center of mass of the Signature motif from the opposite NBD averaged over both domains.

#### Inter-residue distances

The distance between specific pairs of residues was determined in order to compare the simulations to the results from a range of crosslinking studies. The distance between two residues was taken as the distance between the centers of the corresponding Cα atoms, unless otherwise specified. The distance was calculated for each of the configurations stored. The maximum, minimum and average values obtained for 180 ns of the combined trajectory of Runs A and B (inward-facing state) are reported in Table 2 and the combined trajectories of Runs C and D1 to D4 (asymmetric ATP-bound state) are given in Table 3.

**Table 2:** X-linking in the inward-facing ATP-bound state.

Cross-linked residues (human P-gp numbering)			Distance between Cα’s (nm)				
			MD Simulations			Calc. MTS spacer	
helices TM	1	2	Min	Max	Average (2×90 ns)		Ref.
1 & 11	M68C	Y950C	0.8	1.2	1.0 +/– 0.1	0.9 ^a^	[56]
1 & 11	M68C	Y953C	0.9	1.4	1.1 +/– 0.1	0.9^ a^	[56]
1 & 11	M68C	A954C	0.7	1.3	1.0 +/– 0.1	0.9^ a^	[56]
1 & 11	M69C	A954C	0.9	1.5	1.2 +/– 0.1	0.9^ a^	[56]
1 & 12	M69C	L975C	1.0	1.5	1.2 +/– 0.1	0.9 ^a^	[56]
2 & 11	V133C	G939C	0.5	0.7	0.6 +/– 0.1	0.9^ a^	[55]
2 & 11	C137C	A935C	0.5	0.7	0.6 +/– 0.1	0.9^ a^	[55]
3 & 9	L175C	N820C	2.5	3.7	3.2 +/– 0.2	3.0 ^b^	[60]
4 & 12	L227C	S993C	2.4	3.4	2.8 +/– 0.1	0.9^ a^	[63]
4 & 12	V231C	S993C	2.1	2.9	2.5 +/– 0.2	0.9^ a^	[63]
4 & 12	W232C	S993C	1.8	2.8	2.3 +/– 0.2	0.9^ a^	[63]
4 & 12	A233C	S993C	1.6	2.4	2.0 +/– 0.1	0.9^ a^	[63]
4 & 12	I235C	S993C	1.8	2.7	2.2 +/– 0.2	0.9^ a^	[63]
4 & 12	L236C	S993C	1.4	2.5	1.9 +/– 0.2	0.9^ a^	[63]
5 & 12	A295C	S993C	1.0	2.0	1.4 +/– 0.2	0.9^ a^	[63]
5 & 12	I299C	S993C	0.9	2.0	1.4 +/– 0.2	0.9^ a^	[63]
6 & 7	L339C	F728C	1.1	2.0	1.2 +/– 0.1	3.0^ b^	[58]
6 & 7	L339C	A729C	1.1	2.0	1.5 +/– 0.2	3.0^ b^	[58]
6 & 7	F343C	F728C	1.1	2.1	1.5 +/– 0.2	3.0^ b^	[57], [58]
6 & 10	L332C	Q856C	2.3	2.9	2.6 +/– 0.1	2.2 ^c^	[65]
6 & 10	P350C	V874C	2.2	4.1	3.2 +/– 0.4	0.9^ a^	[63]
6 & 10	P350C	E875C	1.8	3.8	2.9 +/– 0.5	0.9^ a^	[63]
6 & 10	P350C	M876C	1.7	3.9	2.9 +/– 0.5	0.9^ a^	[63]
6 & 12	L332C	L975C	0.9	1.5	1.2 +/– 0.1	2.2 ^c^	[61], [62]
6 & 12	L332C	L976C	1.2	1.8	1.5 +/– 0.1	2.2^ c^	[64]
6 & 12	F343C	V982C	1.5	2.3	1.9 +/– 0.1	3.0^ b^	[58], [64]
6 & 12	F343C	M986C	1.3	2.4	1.8 +/– 0.2	0.9^ a^	[62]
6 & 12	G346C	G989C	1.6	3.0	2.2 +/– 0.3	0.9^ a^	[62]
6 & 12	P350C	S993C	1.4	2.8	2.0 +/– 0.2	0.9^ a^	[62]

**Table 3:** X-linking in the asymmetric ATP-bound state.

Cross-linked residues (human P-gp numbering)			Distance between Cα’s (nm)				
			MD Simulations			Calc. MTS spacer	
TM helices	1	2	Min	Max	Average (2×90 ns)		Ref.
1 & 11	M68C	Y950C	0.8	1.4	1.1 +/– 0.1	0.9 a	[56]
1 & 11	M68C	Y953C	0.8	1.6	1.2 +/– 0.1	0.9 a	[56]
1 & 11	M68C	A954C	0.8	1.6	1.2 +/– 0.1	0.9 a	[56]
1 & 11	M69C	A954C	1.0	1.8	1.3 +/– 0.1	0.9 a	[56]
1 & 12	M69C	L975C	0.9	1.7	1.2 +/– 0.1	0.9 a	[56]
2 & 11	V133C	G939C	0.5	0.9	0.6 +/– 0.1	0.9 a	[55]
2 & 11	C137C	A935C	0.5	1.0	0.6 +/– 0.1	0.9 a	[55]
3 & 9	L175C	N820C	2.4	3.7	3.0 +/– 0.2	3.0 b	[60]
4 & 12	L227C	S993C	2.3	3.9	3.3 +/– 0.2	0.9 a	[63]
4 & 12	V231C	S993C	2.0	3.5	2.9 +/– 0.2	0.9 a	[63]
4 & 12	W232C	S993C	1.6	3.2	2.7 +/– 0.2	0.9 a	[63]
4 & 12	A233C	S993C	1.4	2.9	2.4 +/– 0.2	0.9 a	[63]
4 & 12	I235C	S993C	1.6	3.2	2.5 +/– 0.2	0.9 a	[63]
4 & 12	L236C	S993C	1.3	2.8	2.2 +/– 0.2	0.9 a	[63]
5 & 12	A295C	S993C	0.9	2.3	1.6 +/– 0.2	0.9 a	[63]
5 & 12	I299C	S993C	0.9	2.4	1.7 +/– 0.2	0.9 a	[63]
6 & 7	L339C	F728C	0.9	1.9	1.4 +/– 0.1	3.0 b	[58]
6 & 7	L339C	A729C	0.9	1.9	1.4 +/– 0.2	3.0 b	[58]
6 & 7	F343C	F728C	0.9	2.2	1.5 +/– 0.1	3.0 b	[57], [58]
6 & 10	L332C	Q856C	2.5	3.4	3.1 +/– 0.2	2.2 c	[65]
6 & 10	P350C	V874C	1.9	4.5	3.3 +/– 0.4	0.9 a	[63]
6 & 10	P350C	E875C	1.6	4.1	3.0 +/– 0.4	0.9 a	[63]
6 & 10	P350C	M876C	1.4	4.1	3.0 +/– 0.4	0.9 a	[63]
6 & 12	L332C	L975C	1.1	1.9	1.5 +/– 0.1	2.2 c	[61], [62]
6 & 12	L332C	L976C	1.4	2.2	1.8 +/– 0.1	2.2 c	[64]
6 & 12	F343C	V982C	1.2	2.7	2.2 +/– 0.3	3.0 b	[58], [64]
6 & 12	F343C	M986C	1.0	2.5	2.0 +/– 0.3	0.9 a	[62]
6 & 12	G346C	G989C	1.1	3.3	2.4 +/– 0.4	0.9 a	[62]
6 & 12	P350C	S993C	1.1	3.5	2.6 +/– 0.4	0.9 a	[62]

***NOTE:*** In Tables 2 and 3 the distance between Cα atoms has been estimated as follows: 2× (length of cysteine side chain + distance to spacer) + length of spacer.

a Precise reducing agent not provided. Reducing agents used in this study were N,N’-o-phenylenedimaleimide, N,N’-p-phenylenedimaleimide and 1,6-bismaleimidohexane. The spacer span lengths range from ∼0.3 to ∼1.0 nm.

b Cross-linking was attempted with each of the following spacers: M2M (approximate cross-linking span: 0.52 nm), M3M (∼0.65 nm), M4M (∼0.78 nm), M5M (∼0.91 nm), M6M (∼1.04 nm), M8M (∼1.30 nm), M11M (∼1.69 nm), M14M (∼2.08 nm) and M17M (∼2.47 nm). The distance range given is calculated from the span of the largest and smallest spacer cross-linked.

c Reducing agent is copper phenanthroline. Distance between the S atoms is ∼0.37 nm.

## Results and Discussion

Initially four independent 90 ns MD simulations were performed to examine the conformation of ATP-bound P-gp. In two of these simulations, Runs A and B, ATP bound solely to the Walker A motif of each NBD. No direct contacts were formed between ATP and the Signature motif of the opposite NBD. P-gp adopted a similar overall conformation to that observed in MD simulations in the absence of ATP [9]. This conformation has previously been referred to as the inward-facing ATP-bound state in structural studies [2], [3], [5], [6], or the “loosely bound” state in biochemical investigations [19], [43], [44]. In this manuscript it is referred to as the inward-facing ATP-bound state. The trajectories of Runs C and D1 differed significantly from the trajectories obtained for Runs A and B, where one of the two molecules of ATP bound to the Walker A motif, while the second ATP formed stable contacts with the Signature motif of the opposite NBD. This conformation is referred to as the asymmetric ATP-bound state. These two conformational states will be discussed separately.

### Characterization of an inward-facing ATP-bound state

**Figure 1:**
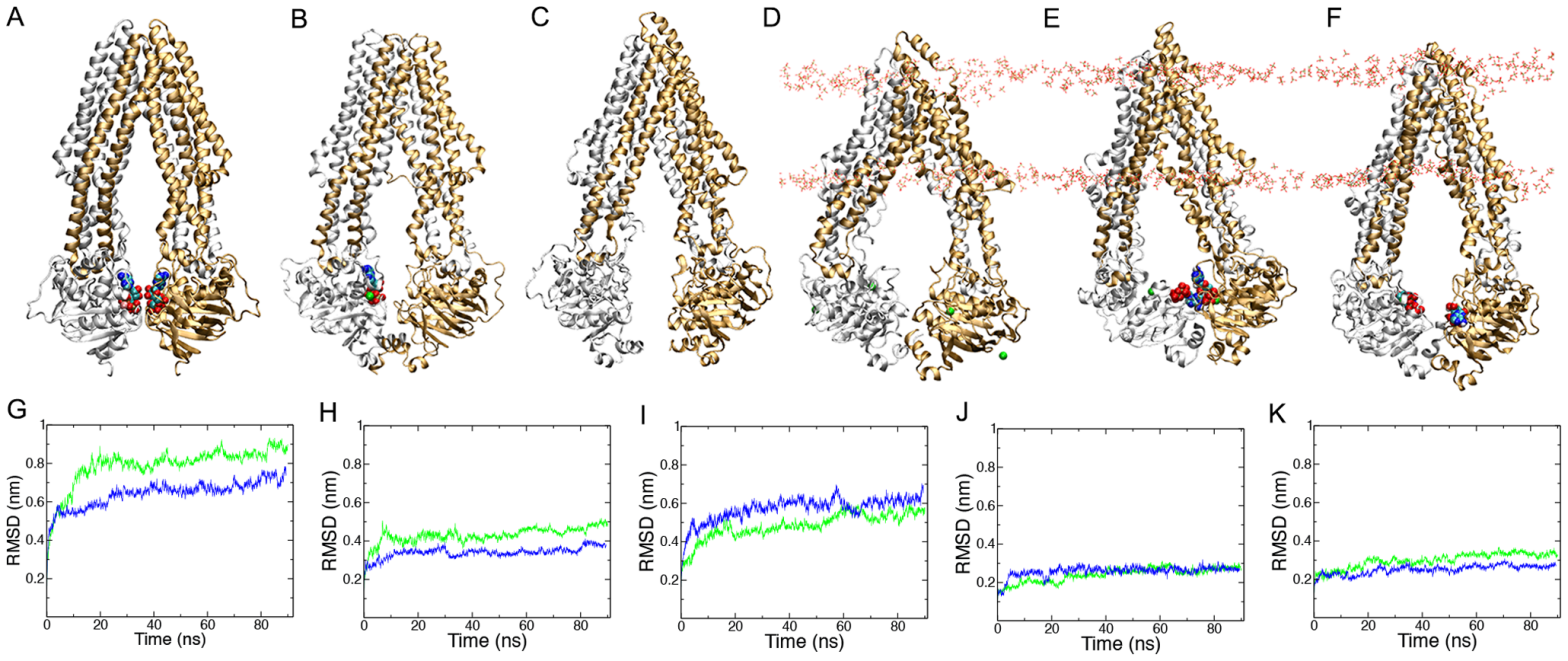
Comparison of the conformation of the inward-open state of P-gp from MD simulations to the crystallographic conformations of homologous ABC transporters. The crystal structures of A) ABCB10 (PDBid 4AYX), B) TM287-288 (PDBid 3QF4) and C) P-gp (PDBid 3G5U). The central conformation of P-gp as determined by cluster analysis D) in the membrane-embedded ATP-free simulations, and from ATP-bound simulations in E) Run A and F) Run B. In each case, the N-terminal and C-terminal halves of each transporter are in gold and silver, respectively. Nucleotide is in CPK colored spacefill and Mg^2+^ ions are shown as green spheres. G) to K) The Cα root mean square deviation (RMSD) of the P-gp simulations from Runs A (green) and B (blue) calculated with respect to the 3G5U-a P-gp crystal structure. The Cα RMSD of G) the P-gp backbone, H) TMD1, I) TMD2, J) NBD1 and K) NBD2.

In the presence of ATP, P-gp rapidly deviated from the crystallographic conformation. In both Run A and Run B, P-gp adopted a conformation similar to that of the XRD structures of TM287-288 [3], ABCB10 [4] and the previously characterized nucleotide-free MD conformation of P-gp [9] within the first 5 ns. The XRD structures of ABCB10, TM287-288 and P-gp [2], the nucleotide-free MD conformation of P-gp, and the most commonly occurring conformation from Run A and Run B are shown in Figures 1A to F, respectively. In the inward-facing ATP-bound conformation observed in Runs A and B, one molecule of ATP binds to each of the Walker A motifs of P-gp. NBD1 and NBD2 pivot towards each other and an interface forms between NBD1 and NBD2 at the C-terminal base of NBDs, similar to that observed in TM287-288 and in the nucleotide-free conformation of P-gp obtained in previous MD simulation studies. The TMD pore of P-gp is accessible from the cytosol, but not from the extracellular environment. After 5 ns of simulation, the Cα root mean square positional deviation (RMSD) considering all Cα atoms in the protein with respect to the P-gp XRD structural model (PDBid: 3G5U, chain A), was 0.53 nm for Run A and 0.56 nm for Run B. The Cα RMSD continued to rise gradually over the remainder of the 90 ns simulation, obtaining values of 0.89 nm in Run A (Figure 1, green) and 0.74 nm in Run B (Figure 1, blue), with respect to the starting model. These values are similar to the Cα RMSD obtained for P-gp in the absence of ATP under the same simulation conditions, where Cα RMSD values of between 0.7 and 1.2 nm were obtained after 60 ns [9]. In large, elongated proteins such as P-gp, the RMSD is highly sensitive to small changes in the relative positions of each domain and to fluctuations in loop regions or movement within helices. This is because minor changes are amplified at the extremities of the protein [9], [38]. For this reason, the Cα RMSD was also calculated separately for each domain of the protein. Figure 1H to K shows the Cα RMSD for TMD1 (residue 42 to 362), TMD2 (residue 709 to 1009), and the RecA-like subdomain of NBD1 and NBD2, respectively. The P-gp NBDs are relatively compact, globular domains consisting of a large RecA-like subdomain, which contains the Walker A and Walker B motifs; and a smaller helical subdomain, which contains the consensus Signature motif for ABC transporters. The relative orientation of these two subdomains is expected to differ between the nucleotide-free and the nucleotide sandwich dimer conformations [45]. The Cα RMSD of the NBD1 RecA-like subdomain (residues 387 to 480 and 547 to 622) was 0.27 and 0.28 nm for Run A and B, respectively. The corresponding values for the NBD2 RecA-like subdomain (residues 1029 to 1114 and 1194 to 1269) were 0.32 and 0.27 nm in Run A and B, respectively. As expected, the Cα RMSD of the elongated α-helical TMDs was higher than that of the globular NBDs. TMD2 had the highest structural flexibility of any individual domain, obtaining Cα RMSD values of 0.58 nm and 0.69 nm after 90 ns of simulation in Runs A and B, respectively. TMD1 was less flexible. After 90 ns of simulation, the Cα RMSD was 0.49 nm in Run A and 0.38 nm in Run B. Note, the magnitudes of the Cα RMSDs for the TMDs and the NBDs are in line with those found in previous simulations of both P-gp and its homologue Sav1866 [9], [46]–[48].

To identify whether the global conformation of P-gp was in fact metastable, the first 10 ns of the trajectories of Run A and Run B were discarded and cluster analysis was performed on all frames contained in the remaining 80 ns using the method of Daura *et al.* [39], [40]. Here conformations of P-gp were considered to be members of the same cluster of conformations if their backbone RMSD was <0.4 nm, as used in previous studies [9]. The central conformations of the dominant clusters from Run A and Run B are shown in Figure 1E and F. In Run A, 96.4% of the conformations of P-gp lay in this dominant cluster during the 80 ns analyzed. In Run B, all conformations were included in the dominant cluster. These results are in contrast to previous studies of nucleotide-free P-gp performed under the same simulation conditions and clustering criteria, which showed that only 20 to 28% of all conformations lay within the dominant cluster [9]. This demonstrates that the inward-facing ATP-bound conformation of P-gp is more structurally stable than a similar conformation of P-gp in the absence of ATP.

In fact, the structures of the central conformation of the Run A and B clusters adopted the same general conformation, but did not belong to the same conformational cluster as identified by our cut-off criteria. The Cα RMSD calculated across all residues of the central structures of Run A and B differed by 0.62 nm. The conformational differences between the two trajectories can be attributed primarily to the relative orientation of the NBDs. This can be quantified in terms of the degree of closure of the nucleotide binding sites, measured as the distance between the center of mass of the Walker B motifs of one NBD and the center of mass of the Signature motif of the opposite NBD. Plots of these distances are given (Figure S1). In both Run A and Run B, the separation between the motifs remained symmetric. Starting from an initial separation of 3.9 nm in the 3G5U-a structure, the distance between the NBD1 Walker B and NBD2 Signature motif forming ATP Binding Site 1 (BS-1), decreased to 3.8 +/– 0.4 and 3.5 +/– 0.2 nm in Run A and Run B, respectively, when averaged over the full 90 ns simulated. The corresponding distance between the NBD2 Walker B and NBD1 Signature motif (BS-2) was initially 3.5 nm. When averaged over the trajectory, this distance decreased to 3.2 +/– 0.3 nm in Run A and 3.2 +/– 0.2 nm in Run B. These values were comparable to those obtained for nucleotide-free P-gp under the same simulation conditions, where the average distance between the center of mass of the Walker B motif and Signature motif was 3.2 +/– 0.7 nm at BS-1 and 3.1 +/– 0.5 nm at BS-2 [9].

**Figure 2:**
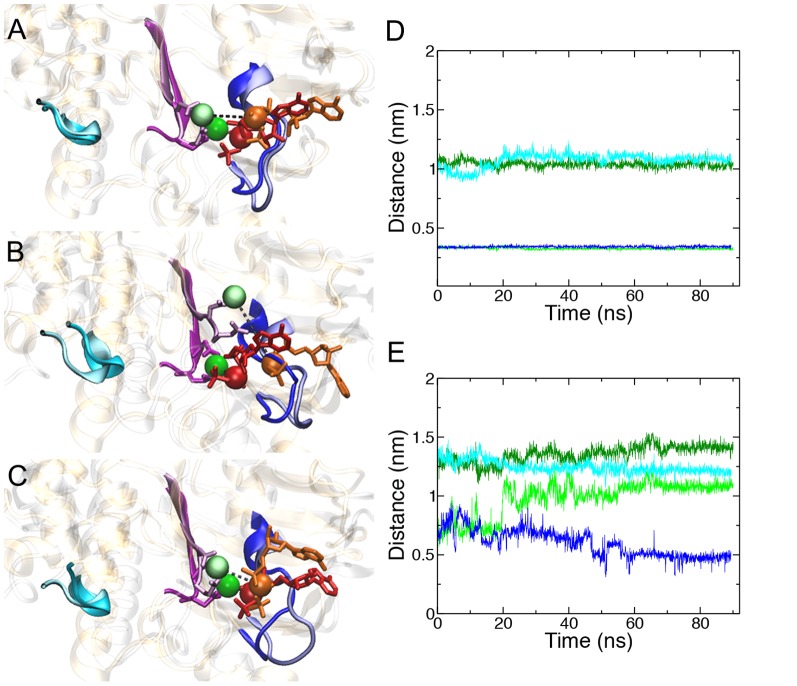
Relative orientation of Mg^2+^ and ATP and in the inward-facing ATP-bound state of P-gp. Overlay of the side view of NBD1 (gold) and NBD2 (silver) with bound ATP and Mg^2+^ in A) the initial conformation, the central conformation from B) Run A and C) Run B. The Walker A (blue), Walker B (purple) and Signature motifs (cyan) are shown as ribbons. The Walker B Asp and Glu are shown in licorice representation. Motifs from NBD1 are bold and motifs from NBD2 are pastel. The ATP bound to NBD1 and NBD2 are colored red and orange, respectively. The ATP β-phosphate is in spacefill. Mg^2+^ bound to NBD1 and NBD2 is dark green and light green, respectively. The distance between the center of mass of Mg^2+^ and the ATP β-phosphate pair is shown as a dashed line. Plots of the Mg^2+^ and β-phosphate distance over time (Run A is green, Run B is blue) and the Cα Walker A Gly and Walker B Asp (Run A: dark green, Run B: cyan) for D) NBD1 and E) NBD2.

Simulations of the stable inward-facing ATP-bound state showed key differences between NBD1 and NBD2 in the manner through which Mg^2+^, ATP and the Walker A and B motifs interact. More importantly, these differences persisted throughout the simulations. Crystallographic studies of isolated ABC transporter NBDs have consistently shown that the Mg-nucleotide complex is coordinated such that the phosphate backbone of ATP simultaneously interacts with the backbone of the Walker A motif and with Mg^2+^, while the Walker B aspartate and adjacent glutamate form the binding site for Mg^2+^. This overall coordination geometry was maintained throughout Runs A and B. Subtle structural and positional differences were nonetheless noted between NBD1 and NBD2. These are shown in Figure 2A to C, which shows the superposition of NBD1 and NBD2. In NBD1 the α- and β-phosphate groups of ATP (red) bound to the backbone of the Walker A residues Gly428 to Thr431 (blue cartoon), while the position of the ATP ribose moiety fluctuated considerably throughout the simulations. Mg^2+^ (green) bound to the Walker B (purple cartoon) Asp551 and adjacent Glu552. In NBD2 the corresponding protein-ligand interactions were maintained. Here the ATP α- and β-phosphate groups (orange) bound the Walker A residues Gly1071 to Thr1074 (pastel blue) and Mg^2+^ (light green) bound to the Walker B Asp1196 and adjacent Glu1197 (pastel purple). In NBD1, Mg^2+^ maintained a direct contact with the β- and γ-phosphate groups of ATP throughout Runs A and B, in line with the crystallographic models [49], [50]. The distance between the center of mass of Mg^2+^ and that of the ATP β-phosphate was 0.33 +/– 0.01 and 0.34 +/– 0.01 nm in NBD1 of Runs A and B, respectively. In contrast, the contact between Mg^2+^ and ATP was not maintained in NBD2 throughout the simulations. The initial distance between the center of mass of Mg^2+^ and that of the ATP β-phosphate was 0.61 nm in NBD2. In Run A, this value was maintained for the first 20ns of simulation before rising rapidly to 1.10 nm and remained at this value for the duration of the simulation. In Run B, the initial distance increased to a maximum of 0.94 nm after 4.1 ns, before slowly decreasing to a distance of 0.50 nm after 90 ns of simulation. To examine whether this motion was associated with structural changes within the ATP binding site itself, the distance between the center of mass of the Cα atom of the Walker B aspartate and that of the Walker A glycine (426 in NBD1 and 1069 in NBD2) was calculated. In the P-gp crystal structure, the distance between these two residues was 1.1 nm in NBD1. Throughout the simulations the distance between the NBD1 Walker B aspartate and Walker A glycine remained constant, with values of 1.1 +/– 0.3 and 1.1 +/– 0.6 nm in Run A and B, respectively. The initial distance between the NBD2 Walker B aspartate and the Walker A glycine was 1.2 nm. This distance increased slightly during the initial phase of the simulation before plateauing at an average value of 1.3 +/– 0.7 nm in Run A and 1.3 +/– 0.5 nm in Run B, as shown in Figure 2. These differences suggest that the ATP binding motifs of NBD2 have a higher degree of flexibility than those of NBD1, and indicate an underlying difference in the coordination of Mg^2+^ and ATP.

The change in the relative orientation and the internal flexibility of the NBDs was accompanied by small displacements in the relative positions of the TM helices when compared to the starting 3G5U XRD structural model, as can be seen in Figure 1. During the 90 ns simulations of Run and B, kinks appear in the membrane-embedded span of five of the twelve TM helices: TM1, TM6, TM7, TM9 and TM12. These kinks are induced by the presence of Pro65 (TM1), Pro346 (TM6), Pro705 (TM7), Pro722 (TM7) and Pro992 (TM12). Two additional proline residues are present in the membrane-embedded span of the TMD helices, Pro219 and Pro862, at pseudo-symmetrical positions in TM4 and TM10. The presence of proline within an α-helix is known to disrupt the α-helical hydrogen bonding pattern and alters the torsional angle of the preceding residue. Previous studies have shown that a proline residue induces a kink in α-helix with an average angle of 21 +/– 11° [51], [52]. Such proline induced kinks within transmembrane domains are believed to play an important role in the propagation of conformational changes [51].

Analysis of the conformations of TMD1 and TMD2 shows that there is a narrowing of the two proposed substrate uptake portals. In the 3G5U structure, these portals lie in the membrane-embedded region of the TMDs, forming two lipid-accessible clefts on opposite sides of the protein. The proposed portals are located between TM helices 3, 4 and 6 and between TM helices 9, 10 and 12 respectively [2]. There was a gradual decrease in the total solvent accessible surface area (SASA) of the membrane-embedded TM3, 4 and 6 portal and TM 9, 10 and 12 portal throughout the simulations. The TM3, 4 and 6 portal had an initial total SASA of 29.9 nm^2^, which decreased to an average value of 16.2 +/– 0.3 nm^2^ after 90 ns in Run A and Run B. This decrease can be attributed to a significant decrease in the hydrophobic component of the SASA, dropping from an initial value of 20.3 nm^2^ to 9.9 +/– 0.3 nm^2^ after 90 ns. This was associated with the narrowing of the TM3, 4 and 6 portal. A marked decrease in the total and hydrophobic SASA is also observed at the TM9, 10 and 12 portal. The total SASA of the TM9, 10 and 12 portal is 37.73 nm^2^ in the membrane-embedded 3G5U P-gp. After 90 ns, the total SASA of the TM 9, 10 and 12 portal had decreased to 23.3 +/– 1.6 nm^2^, when averaged across Run A and B, significantly greater than that of the TM 3, 4 and 6 portal. This again resulted primarily from a decrease in the hydrophobic SASA. The hydrophobic SASA of Run A and B decreased from an initial value of 23.3 nm^2^ to obtain an average value of 13.4 +/– 1.5 nm^2^ after 90 ns. Plots of the total, hydrophobic and hydrophilic SASA are given (Figure S2). This relative closure of the TM portals is in contrast to previous investigations of nucleotide-free P-gp, in which the SASA of both portals remained essentially the same as that of the 3G5U-a P-gp crystal structure [9], indicating that the relative closure of the TM portals is induced by the presence of ATP.

### Spontaneous formation of an asymmetric ATP-bound state

In Runs C and D1, P-gp underwent a large-scale conformational change in the presence of Mg^2+^ and ATP. The most notable characteristic of this conformational change was the direct interaction of NBD1 and NBD2 with ATP at Binding Site 2 (BS-2), formed by the Walker A motif of NBD2 and the Signature motif of NBD1. Here, the ATP at BS-2 formed a direct contact with the Signature motif of NBD1, inducing the formation of a subunit interface between the NBD2 RecA-like subdomain and the NBD1 helical subdomain, with ATP tightly bound between them. This tight binding interface formed within the first 30 ns of both Runs C and D1. In order to increase the sampling of the conformational space of the protein and observe whether a similar conformation would evolve at BS-1, the conformation of P-gp from Run D1 after 30 ns of simulation was used as the starting conformation for three additional simulations. Run D1 was chosen for this purpose, as the orientation of the NBDs appeared closer to a nucleotide sandwich dimer, in which both ATP molecules were tightly bound to the NBDs, than in Run C. Note, new velocities were assigned to each of the branched simulations, denoted Run D2, D3 and D4.

**Figure 3:**
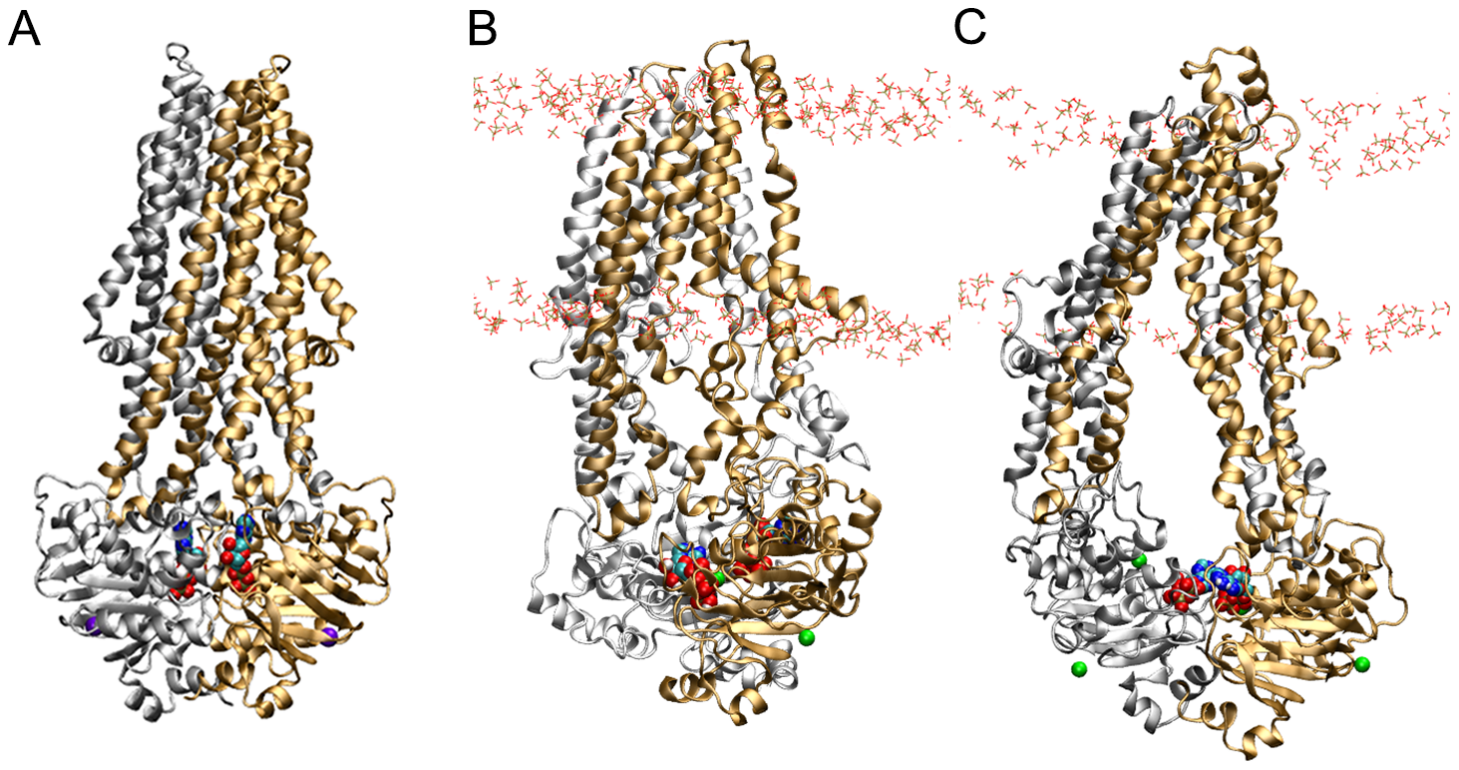
Comparison of the P-gp asymmetric ATP-bound state to ADP-bound Sav1866. Front view of A) the ADP-bound XRD structure of Sav1866, the central conformation of B) Run 4, and C) the branched D series simulations obtained from cluster analysis. The N- and C-terminal halves of each transporter are in gold and silver, respectively. Mg^2+^ is shown as green spheres and ATP is in CPK coloring. The phosphate groups of the lipid headgroups are shown in licorice.

Cluster analysis was performed on the combined trajectory of Runs D1 to D4. Despite new velocities being assigned to each simulation, cluster analysis revealed that P-gp adopted a metastable state with single dominant conformation throughout 65% of the combined trajectory, or 254 of the 360 ns considered. Throughout the combined Run D series trajectory, 10 transitions occurred between this dominant cluster and a secondary conformational cluster, which was populated for 18% of the trajectory, or 70 ns of the 360 ns considered. The conformations of the dominant cluster and the secondary cluster were very similar: the Cα RMSD between the central structures of the dominant and secondary clusters was only 0.41 nm, just outside the 0.40 nm cut-off for members of the same cluster, indicating that the series of trajectories that correspond to Run D captured a metastable intermediate. Cluster analysis of the conformation of P-gp from the 120 ns trajectories of Runs C and D1 showed that the conformation of P-gp differed between the two non-branched simulations. Nevertheless, in each simulation the protein adopted a stable, dominant conformation. In Run C, the structure of P-gp lay within the dominant cluster of conformations for 62% of the 120 ns trajectory while in Run D1, the structure of P-gp occupied the dominant conformational cluster for 83% of the 120 ns trajectory. Figure 3 shows the central structure of the dominant conformational cluster from Run C and D1. The conformational differences between the trajectories of Run C and the D series simulations were primarily due to positional differences in the TMDs and ICLs, which dominates the RMSD in this highly elongated protein [38].

**Figure 4:**
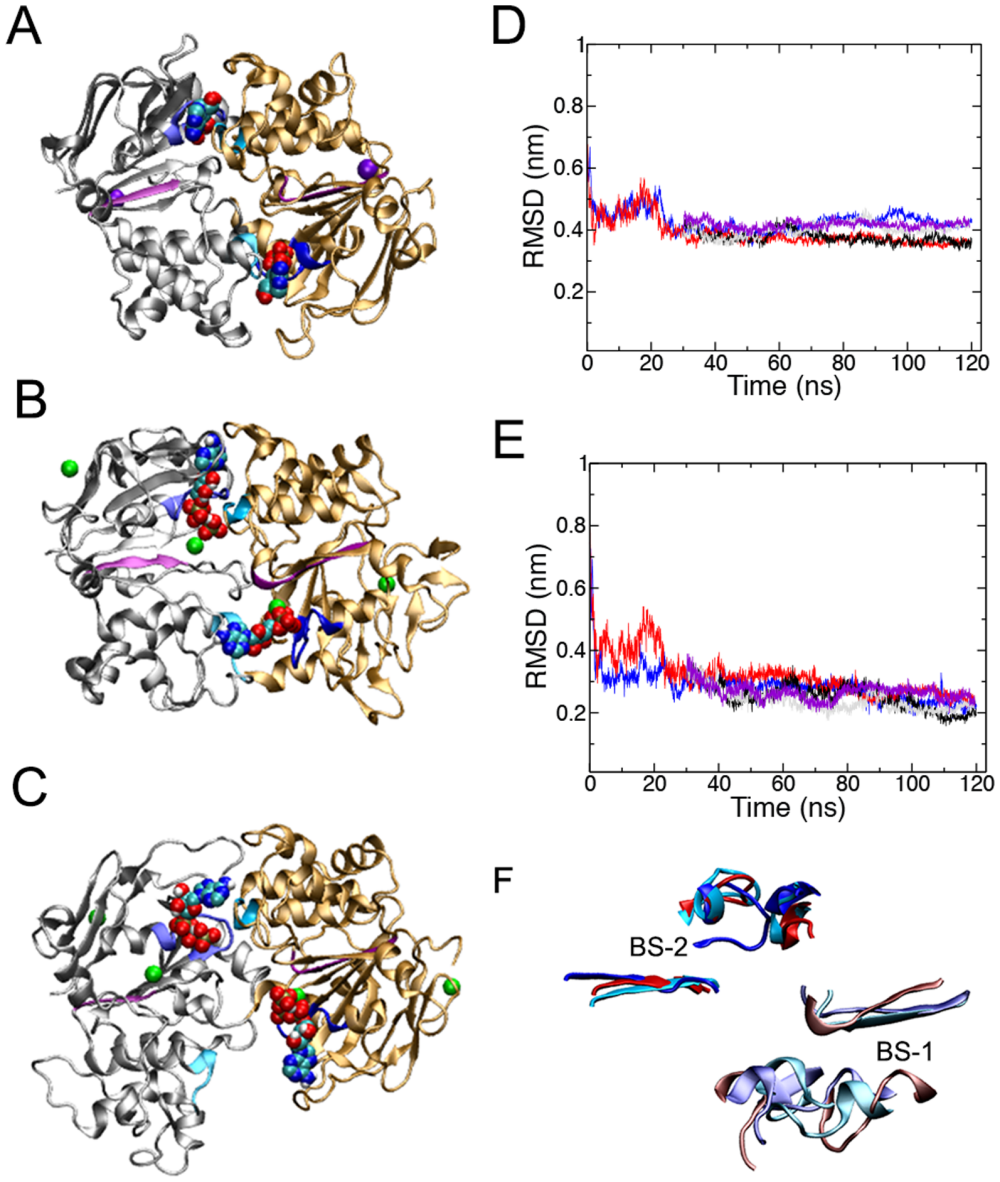
Comparison of the NBD conformation of the asymmetric ATP-bound state to the canonical “nucleotide sandwich dimer”. Top view of the NBDs of A) ADP-bound Sav1866, and the central conformation of the dominant cluster from B) Run C, and C) the Run D series simulations. Cα RMSD of D) the entire NBD1 and the NBD2 Rec-A like subdomain; and E) binding site 2, composed of the NBD2 Walker A and B motifs and NBD1 Signature motif, from Runs C and D1 to D4 calculated with respect to the ADP-bound Sav1866 XRD structure. F) Superposition of binding site 1 and 2 (BS-1 and BS-2) from Sav1866 (cyan), and the central conformation of Run C and the Run D series simulations obtained from cluster analysis.

In both Run C and the D series simulations, the formation of a tight NBD-NBD interface at ATP BS-2, formed by the NBD2 Walker B and NBD1 Signature motif, induced an asymmetric orientation of the NBDs. This is shown in Figures 3 and 4, and is evident in both the degree of closure of the two ATP binding sites and the relative binding and orientation of ATP at each binding site. The distance between the center of mass of the Walker B motif and the center of mass of the Signature motif was 2.2 +/– 0.3 nm at BS-2 when averaged across the original Runs C and D1 and Runs D2 to D4. This value is 1.0 nm less than the 3.2+/–0.3 nm obtained in the inward-facing ATP-bound conformation of P-gp, demonstrating a significant closure of the NBDs at BS-2. At BS-1, the corresponding distance between the center of mass of the NBD1 Walker B motif and NBD2 Signature motif decreased from an initial value of 3.9 nm to 3.0 +/– 0.4 nm, when averaged across the Run C and D1 to D4 simulations. This value was again significantly less than that of the inward-facing ATP-bound conformation (3.7 +/– 0.3 nm). Plots of these distances are given (Figure S3), and the initial, final and average values for each simulation are given in Table 1. When taken together, these simulations show an asymmetric closure of the NBDs and the dimerization of NBD1 and NBD2, with bound ATP sandwiched between them at BS-2, but the NBDs did not adopt the canonical nucleotide sandwich dimer conformation. Note, it has been demonstrated experimentally that P-gp retains its transport and ATPase activity in the absence of the subunit linker [53], therefore it is unlikely that conformation observed in Run C and D can be attributed to the lack of the subunit linker region in MD simulations. Furthermore, these results suggests that after 120 ns of simulation, the NBD conformation in Run C is the closest to forming a nucleotide sandwich dimer, characterized by close contacts for ATP at both ATP binding sites.

In order to quantify how far the trajectories of the asymmetric state of P-gp deviated from the canonical nucleotide sandwich dimer observed in the ADP-bound XRD structure of Sav1866 (PDBid: 2HYD) [1], the entire NBD1 and the NBD2 RecA-like domain of the Run C and D series of simulations were fitted to the corresponding residues in the Sav1866 XRD structural model. The Cα RMSD was then calculated throughout the trajectories. At the start of the simulation period, the Cα RMSD was 0.66 nm. Figure 4D shows that the Cα RMSD decreased over the first 25 ns of simulations and then plateaued. The final value, averaged over the final frames of the Runs C and D1 to D4, was 0.40 +/– 0.03 nm. As mentioned above, the ATP bound to BS-2 forms direct interactions between both the BS-2 Walker A and Signature motif. Comparison of the Cα RMSD of the isolated BS-2 Walker A and Signature motifs from the Runs C and D series of simulations after fitting them to the ADP-bound Sav1866 conformation showed the initial Cα RMSD was 0.74 nm. During all simulations, the Cα RMSD of the BS-2 motifs decreased steadily (Figure S4). The final value was 0.22 +/– 0.02 nm, averaged over the final frames of each of the five trajectories. Figure 4F shows that the final conformation of BS-2 is consistent with that observed in Sav1866.

**Figure 5:**
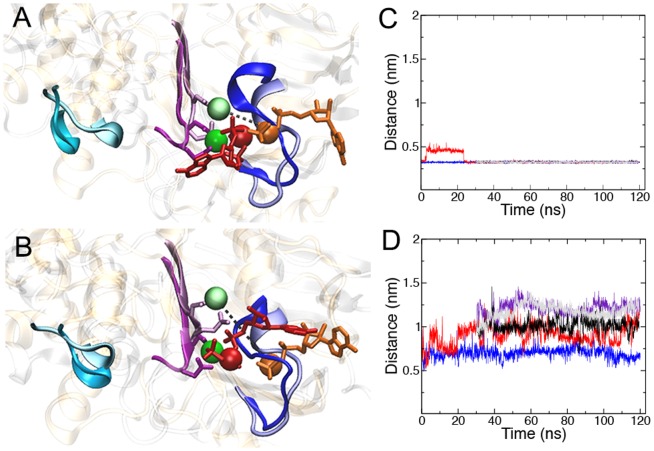
Relative coordination of Mg^2+^ and ATP in the asymmetric ATP-bound state of P-gp. Side view of NBD1 (gold) and the superimposed NBD2 (silver) the central conformation from A) Run C and B) the branched D series simulations as determined by cluster analysis. The bound ATP and Mg^2+^ is shown for both NBDs. The Walker A (blue), Walker B (purple) and Signature motifs (cyan) are shown as ribbons. The ATP bound to NBD1 and NBD2 are red and orange licorice representations, respectively. The ATP β-phosphate is in spacefill. Mg^2+^ bound to NBD1 and NBD2 are dark and light green, respectively. The Walker B Asp and Glu are shown in licorice representation. Motifs from NBD1 are bold and motifs from NBD2 are pastel. The distance between the center of mass of Mg^2+^ and the ATP β-phosphate pair is shown as a dashed line. C) & D) Plot of the Mg^2+^ and β-phosphate distance over time for the original Runs C and D1 to D4 and the three branched D series simulations for C) NBD1 and D) NBD2.

That the two ATP binding sites may not be structurally equivalent has been suggested previously [19]. For example, Senior et al. proposed that ATP hydrolysis might occur at alternating sites, implying the structure of the protein was asymmetric [18]. It has also been demonstrated experimentally that the NBDs of P-gp differ in their binding affinities for both Mg^2+^ and ATP. This would suggest there are structural differences in the coordination of Mg^2+^ and ATP at NBD1 and NBD2 [54]. However, crystallographic studies of ABC transporter NBDs have failed to identify any easily quantifiable difference that could account for the variation in binding affinity. Figure 5 shows that differences in the binding and coordination of Mg^2+^ and ATP exist between NBD1 and NBD2 in the asymmetric ATP-bound conformations of P-gp. Analysis of Runs C and D1 to D4 showed that the NBD1 Walker B Mg^2+^ and the center of mass of the ATP β-phosphate maintained an average distance of 0.32 +/– 0.01 nm throughout Runs C, D2, D3 and D4, indicating that Mg^2+^ formed a direct contact with the ATP phosphate moieties throughout these simulations. It should be noted that in Run D1, this distance initially increases to 0.46 +/– 0.02 nm between 3 and 24 ns of simulation, before adopting an average distance of 0.32 +/– 0.01 nm throughout the remaining 96 ns of simulation, restoring the direct contact between Mg and ATP. Figure 5 shows that, in contrast to NBD1, the direct interaction between Mg^2+^ and ATP was not maintained in NBD2 in Run C or D1 to D4. Instead, the coordination of Mg^2+^ and ATP was consistent with the results of the inward-facing states identified in Runs A and B. The distance between the Mg^2+^ and that of the center of mass of the ATP β-phosphate increased from an initial value of 0.61 nm to an average of 1.18 +/− 0.20 nm across the Run C and Run D series at the end of the simulations. To determine whether this variation was an effect of a conformational change within the vicinity of the ATP binding residues of each NBD, the distance between the center of mass of the Cα of the Walker B aspartate and that of the Walker A glycine (426 in NBD1 and 1069 NBD2) was tracked throughout the Runs C and D1 to D4. The plots are provided (Figure S5). In NBD1, the initial distance between the center of mass of the Walker B aspartate Cα and that of the Walker A glycine (426) of NBD1 was 1.05 nm. This was maintained throughout the five simulations, with an average distance of 1.07 +/– 0.08 nm. In NBD2, the initial value between the corresponding residues was 1.20 nm. The distance increased over the five simulations to an average of 1.30 +/– 0.02 nm in NBD2, suggesting that NBD2 has greater internal flexibility than NBD1.

The crystal structures of the P-gp homologues Sav1866 and MsbA show closure of the TMDs regions proximal to the NBDs and splaying of the extracellular aspects of the TMDs in the nucleotide-bound state [1], [5], [10]. These changes are believed to be associated with the extracellular release of substrate on formation of a “nucleotide sandwich dimer” complex in which two molecules of ATP are bound in a high-affinity conformation between both NBDs [2], [5]. These large conformational differences in the TMDs were not observed in the asymmetric ATP-bound state of Runs C and D1 to D4. Instead, the conformation of the TMDs was consistent with that observed in the inward-facing ATP-bound state of Runs A and B, suggesting that the formation of an asymmetric ATP-bound state is not sufficient to induce a conformational change in the TMDs in the absence of a transport substrate, but produces an asymmetric ATP-bound state in which P-gp is primed for substrate transport and ATP hydrolysis. Our results suggest that either the formation of a nucleotide sandwich dimer, or the binding of a transport substrate to the TMDs is required to induce the conformational changes in the TMDs that lead to the outward-facing conformation believed to be associated with the transport of substrate.

### Comparison to experimental cross-linking data

Several experimental groups have introduced pairs of cysteine residues at specific locations in the TMDs and ICLs of cysteine-free isoforms of P-gp in order to perform cross-linking studies. In these studies, ATP or a non-hydrolysable analogue is added and allowed to bind to P-gp prior to the addition of chemical reducing agents of different lengths. These agents promote the formation of a covalent bond between the sulfur atom of the two cysteines and the reducing agent itself. If P-gp retained the ability to hydrolyze ATP after the covalent modification of the cysteines had occurred, the length of the spacer was considered to be compatible with the physiological ATP-bound conformation of P-gp, allowing the minimum distance between the Cα atoms of the two residues to be inferred. In order to verify whether the inward-facing ATP-bound conformation and the asymmetric ATP-bound conformations of P-gp were compatible with the cross-liking data, the distances inferred from such chemical cross-linking studies were compared to the distances measured between Cα atoms of the corresponding residue pairs from the simulations. For each conformation, the simulation trajectories were concatenated and the minimum, maximum and average distance between pairs of residues was determined as described in the Methods.

**Figure 6:**
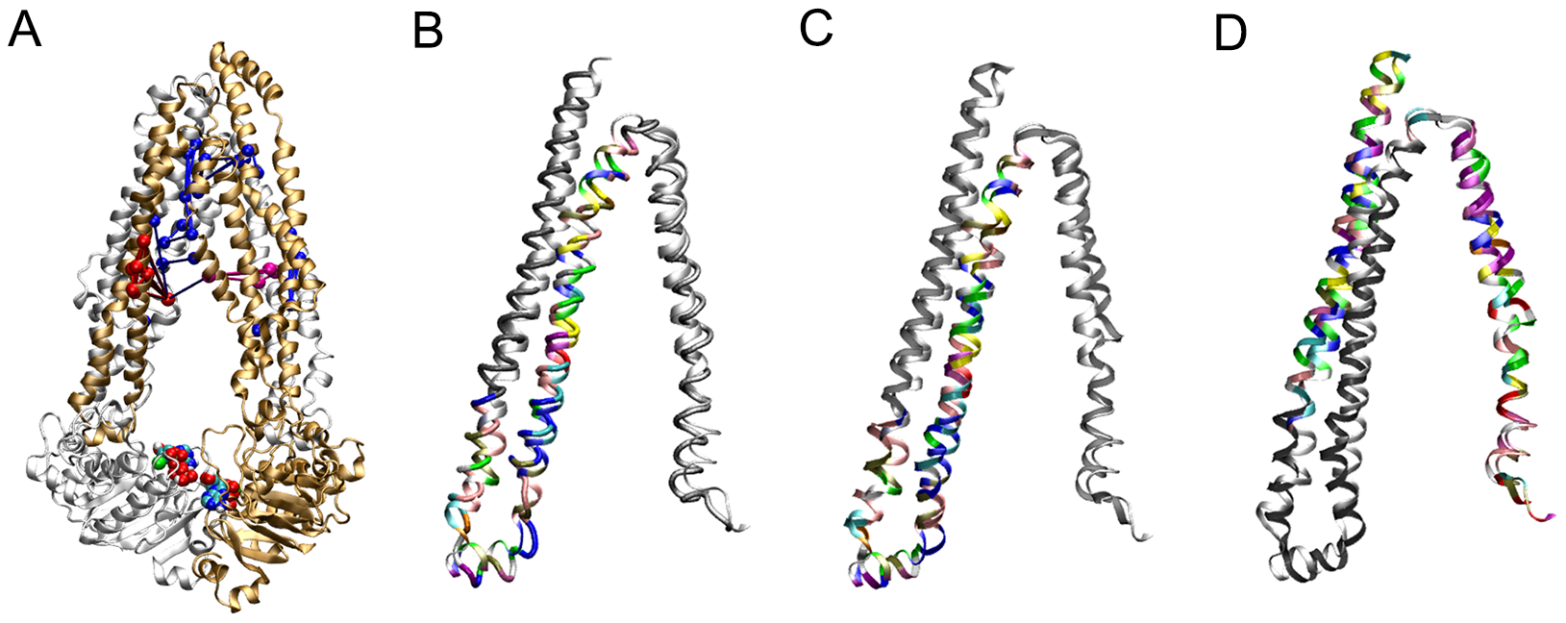
Comparison of the inward-facing ATP bound state to the experimental data. A) Spatial mapping of the cross-linked residues onto the central conformation of Run B from cluster analysis. The N-terminal and C-terminal halves of P-gp are shown in gold and silver, respectively. ATP and the Cα’s of the cross-linked residues are shown as spheres. Residue pairs that do not satisfy the spatial constraints are shown in pink (TM6 and TM10 cross-links) or red (TM4 and TM12 cross-links). B) Superposition of the 3G5U-a (tube) C) and D) 4M1M-a (embossed pastel ribbon) structures with P-gp (bright ribbon). In B) and C), TM4 is colored by residue name. In D), TM3 and 5 are colored by residue name.

Previous mutagenesis studies have identified 29 pairs of residues in the TMDs and ICLs of P-gp that were able to be cross-linked in the presence of ATP or a nucleotide analogue [55]–[65]. The spatial mapping of these 29 pairs of cross-linked residues onto the central conformation of Run B is shown in Figure 6A. The minimum distance obtained in the simulations was less than or equal to the distance calculated from cross-linking studies in 14 of the 29 pairs identified for the inward-facing ATP-bound conformation. For a further 6 pairs, the minimum distance obtained from simulations of the inward-facing ATP-bound conformation was within 0.5 nm of the calculated distance, giving a total of 20 of the 29 pairs that were satisfied or nearly satisfied by the inward-facing ATP-bound conformation. The asymmetric conformation satisfied the same 14 pairs as the inward-facing ATP bound conformation, plus 1 additional pair. If distances within 0.5 nm of the calculated distance were also considered, an additional 8 cross-links could be satisfied or nearly satisfied in the asymmetric ATP-bound conformation, giving in total 23 of the 29 cross-linking pairs.

In 8 of the 9 remaining pairs in the inward-facing ATP-bound conformation, and the remaining 6 pairs in the asymmetric ATP-bound conformation, the distance measured in simulations was not compatible with the cross-linking data. Figure 6A shows that in these cases, one of the residues in the pair lies in TM4 or TM6, and in particular, cross-links between residue 993 of TM12 and the TM4 residues 227 to 236 (pink); and between residue 350 from TM6 and residues 874, 875 and 876 of TM10 (red), show large deviations. Interestingly, these regions are located within the span of the inner leaflet of the membrane, away from the regions predicted to undergo the largest structural changes when based on the comparison of crystallographic data from nucleotide-free P-gp and nucleotide-bound Sav1866. This predicted opening of the TMDs was not observed here. Furthermore, it should be noted that in the MD simulations presented here, the distances between residue pairs located towards the extracellular side of the TMDs fulfill the cross-linking data.

Previous MD simulations of nucleotide-free P-gp (PDBid 3G5U) also noted discrepancies between the cross-linking distances involving TM helix 4 and 6 and the calculated distances from both the 3G5U P-gp structure and MD simulations. This work suggested that the position or orientation of TM helix 4 and TM helix 6 is inappropriate in the 3G5U crystal structure. [9] Our current MD simulations of ATP-bound P-gp also support this conclusion. That the positions of several helices in 3G5U is inappropriate has been confirmed in the 2013 XRD structural models of mouse P-gp determined by Ward *et al.* (PDBid 4KSB) [7] and the SAD refined XRD structural model of mouse P-gp determined by Li *et al.* (PDBid 4M1M) [8]. Both of the 2013 structures contain differences in the backbone position of TM4, resulting a vertical displacement of the TM4 helix, and register shifts of one amino acid in TM3 and TM5 when compared to the 3G5U P-gp structure. These positional displacements and register shifts affect the relative position of the transmembrane helices within the span of the lipid membrane, and also influence the helix-helix interactions with neighboring helices prior to MD equilibration. To determine whether the simulations were biased by the choice of starting structures, or converged to the refined structures, the 3G5U and 4M1M P-gp structures and the central conformation of Run B were superimposed and the residue orientation in TM3, 4 and 5 was analyzed. The central conformation of Run B was chosen, as it most closely resembles the conformation of the 3G5U and 4M1M crystal structures. Figure 6 shows that there is a vertical displacement of the TM4 helical backbone in simulations (ribbon) when compared to the 3G5U structure (tubes), providing a close match to the vertical displacement observed in the 4M1M structure (embossed pastel ribbon) and correcting the apparent register shift observed in Figure 6B when compared to the starting 3G5U conformation. In particular, the unstructured region of TM4 that leads into the TM4-TM5 intracellular loop adopts an α-helical conformation in simulations, similar to that observed in the 4M1M structure (Figure 6C). Li *et al*. has also reported several other changes in the refined 4M1M P-gp structure when compared to the original 3G5U structure, including a register shift of 1 amino acid in TM3 and TM5 [8]. Comparison of the residue distribution of the central conformation Run B and the 4M1M structure (Figure 6D) show an agreement in the helical register of TM3 and a register match within the majority of TM5, indicating that our simulations are: a) not heavily influenced by the use of the 3G5U-a P-gp structure as the starting conformation; and b) reproduce the main structural differences between 3G5U and 4M1M.

When considering the reproduction of experimental data, it is important to note that the experimental cross-linking studies involved timescales of minutes, and the experiments were conducted at a range of temperatures and experimental conditions to promote the formation of cross-links. In contrast, the conformations sampled from MD simulations correspond to a nanosecond timescale. In addition, on the timescales of the simulations it was not possible to capture the canonical nucleotide sandwich dimer conformation that is believed to represent the conformation in which ATP-bound cross-linking data is collected. Finally it must be remembered that the structures proposed based on X-ray diffraction data represent a time and ensemble average. In addition, the data on which these structures are based has been symmetrized and correspond to the molecule in a highly ordered crystalline environment in the presence of detergent. In contrast, the configurations analyzed from the simulations are individual snapshots in time. The incorporate both thermal motion at 300K and the range of states accessible to the system in a membrane environment.

## Conclusion

These simulations capture two metastable conformations of ATP-bound P-gp, suggesting that P-gp exists in a spectrum of stable ATP-bound conformations under physiological conditions. The two conformational states characterized here corresponded to an inward-open ATP-bound state in which one molecule of ATP was bound solely to the Walker A motif of each NBD; and an asymmetric ATP-bound state, where one ATP was bound solely to the Walker A motif of NBD1 and one ATP was bound simultaneously to the NBD2 Walker A and the NBD1 Signature motif. Both the inward-facing and asymmetric ATP-bound states were demonstrated to be structurally stable over 90 to 120 ns. The inward-facing state of ATP-bound P-gp is consistent with that observed in the XRD structures of ABCB10 and TM287-288, and the conformation of membrane-embedded nucleotide-free P-gp predicted in previous simulation studies [9]. In fact, when compared to previous simulations of P-gp in the absence of ATP [9], the addition of ATP enhanced the stability of the membrane-embedded P-gp structure. Both the inward-facing and asymmetric ATP-bound states showed a consistent set of differences in the interaction and orientation of ATP and the catalytic Mg^2+^ bound at the Walker B motif of each NBD. These most likely arise from subtle structural asymmetries in the relative distances between the Walker A and Walker B motifs. These small structural asymmetries are present in the P-gp crystal structure and become enhanced in MD simulations. We propose that these differences in the coordination of Mg^2+^ and ATP may regulate the formation of a contact interface between ATP bound at the NBD1 Walker A motif and the NBD2 Signature motif, and thus the rate of hydrolysis of ATP.

## Supporting Information

Figure S1 **Separation of the ATP binding sites in the inward-facing P-gp simulations.** The distance between the center of mass of A) the NBD1 Walker B motif and NBD2 Signature motif; and B) the NBD2 Walker B motif and NBD2 Signature motif of Run A and B as a function of simulation time. Run A is green and Run B is blue.
(TIF)

Figure S2 **Solvent accessible surface area (SASA) of the TM3, 4 and 6 portal and TM9, 10 and 12 portal.** The total and hydrophilic SASA of the A) TM3, 4 and 6 portal, and B) TM9, 10 and 12 as a function of simulation time. Run A is shown in dark green (total SASA) and light green (hydrophilic SASA) and Run B is shown in blue (total SASA) and cyan (hydrophilic SASA).
(TIF)

Figure S3 **Separation of the ATP binding sites in the asymmetric ATP-bound P-gp simulations.** The distance between the center of mass of A) the NBD1 Walker B motif and NBD2 Signature motif; and B) the NBD2 Walker B motif and NBD2 Signature motif of Run C (blue) D1 (red), D2 (purple), D3 (black) and D4 (grey) as a function of simulation time.
(TIF)

Figure S4 **Binding of ATP to the Signature motif in the asymmetric ATP-bound simulations.** The distance between the center of mass of the ATP β phosphate and that of the Signature motif serine Cα in the asymmetric ATP-bound P-gp simulations in A) ATP Binding Site 1, and B) ATP Binding Site 2 of the Run C (blue), D1 (red), D2 (purple), D3 (black) and D4 (grey) simulations.
(TIF)

Figure S5 **Distance between the Walker A and B motifs in the asymmetric ATP-bound P-gp simulations.** The distance between the centre of mass of the Cα of the Walker B aspartate and that of the Walker A glycine of A) NBD1 (residue 426) and B) NBD2 (residue 1069) calculated throughout the MD trajectories of Run C (blue) D1 (red), D2 (purple), D3 (black) and D4 (grey).
(TIF)
