# Supplementary figures and images for: Structural Characterization of Two Metastable ATP-Bound States of P-Glycoprotein
Source: PLoS One. 2014 Mar 14;9(3):e91916. doi: 10.1371/journal.pone.0091916 (PMC3954865; doi:10.1371/journal.pone.0091916)

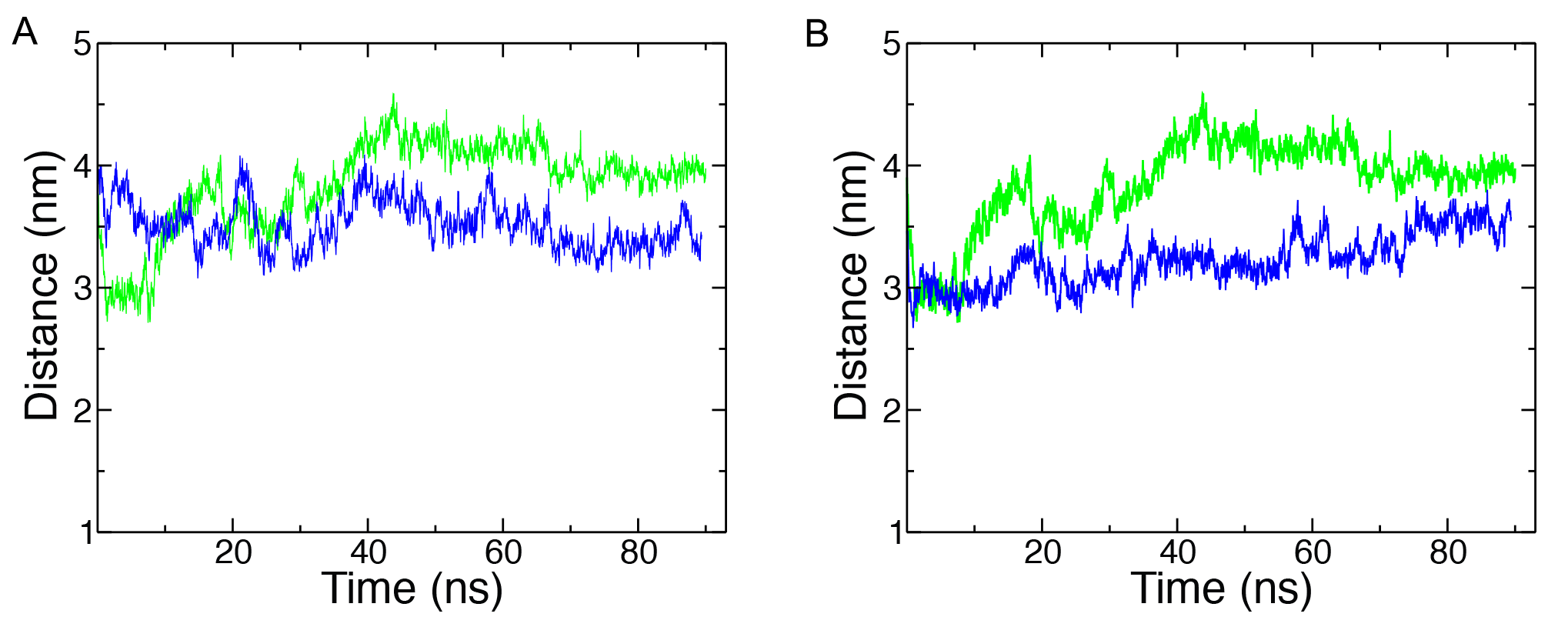

Supplement: Figure S1 — Separation of the ATP binding sites in the inward-facing P-gp simulations. The distance between the center of mass of A) the NBD1 Walker B motif and NBD2 Signature motif; and B) the NBD2 Walker B motif and NBD2 Signature motif of Run A and B as a function of simulation time. Run A is green and Run B is blue. (TIF) [file pone.0091916.s001.tif]

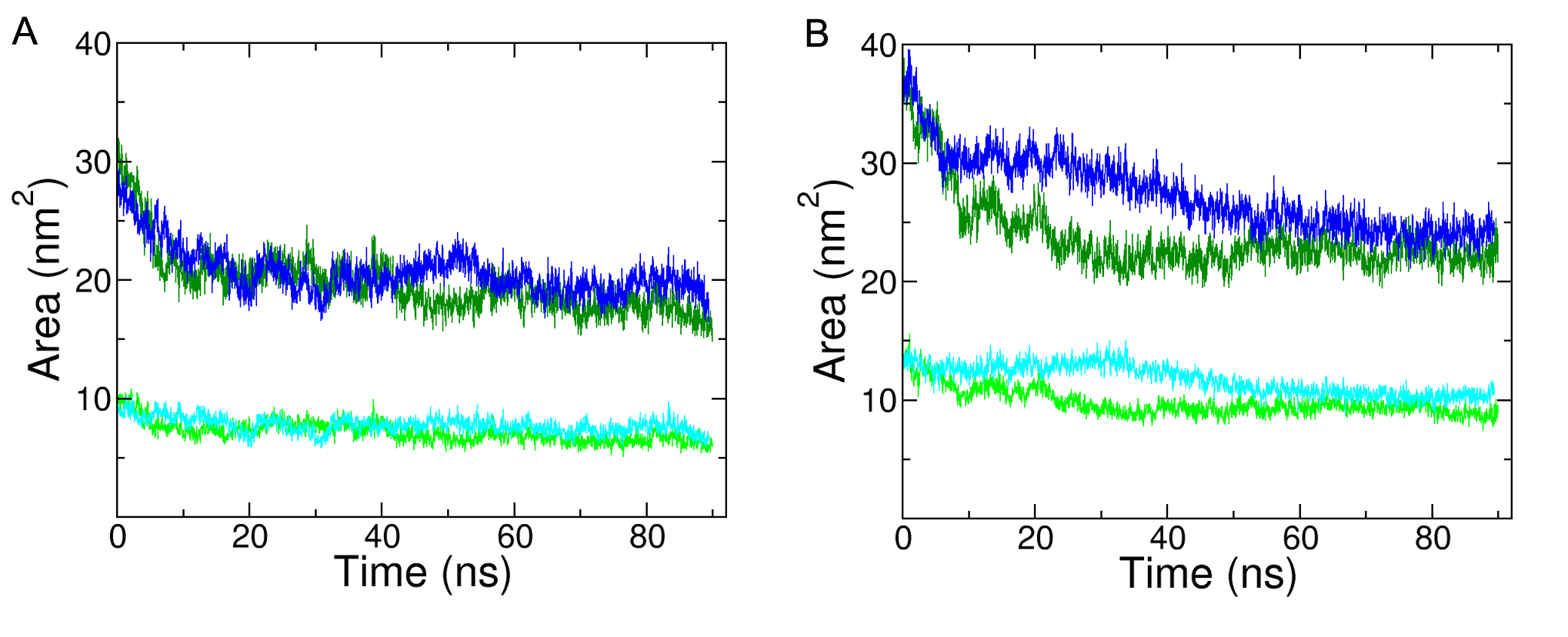

Supplement: Figure S2 — Solvent accessible surface area (SASA) of the TM3, 4 and 6 portal and TM9, 10 and 12 portal. The total and hydrophilic SASA of the A) TM3, 4 and 6 portal, and B) TM9, 10 and 12 as a function of simulation time. Run A is shown in dark green (total SASA) and light green (hydrophilic SASA) and Run B is shown in blue (total SASA) and cyan (hydrophilic SASA). (TIF) [file pone.0091916.s002.tif]

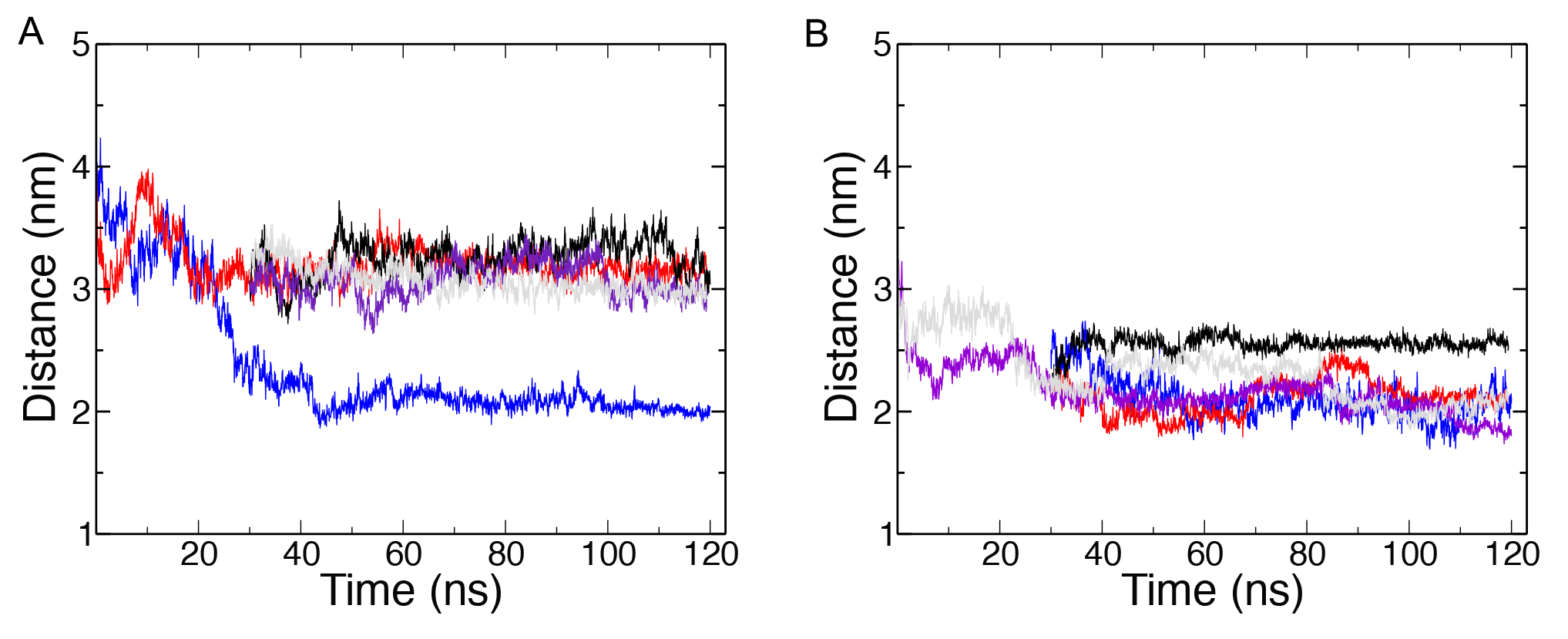

Supplement: Figure S3 — Separation of the ATP binding sites in the asymmetric ATP-bound P-gp simulations. The distance between the center of mass of A) the NBD1 Walker B motif and NBD2 Signature motif; and B) the NBD2 Walker B motif and NBD2 Signature motif of Run C (blue) D1 (red), D2 (purple), D3 (black) and D4 (grey) as a function of simulation time. (TIF) [file pone.0091916.s003.tif]

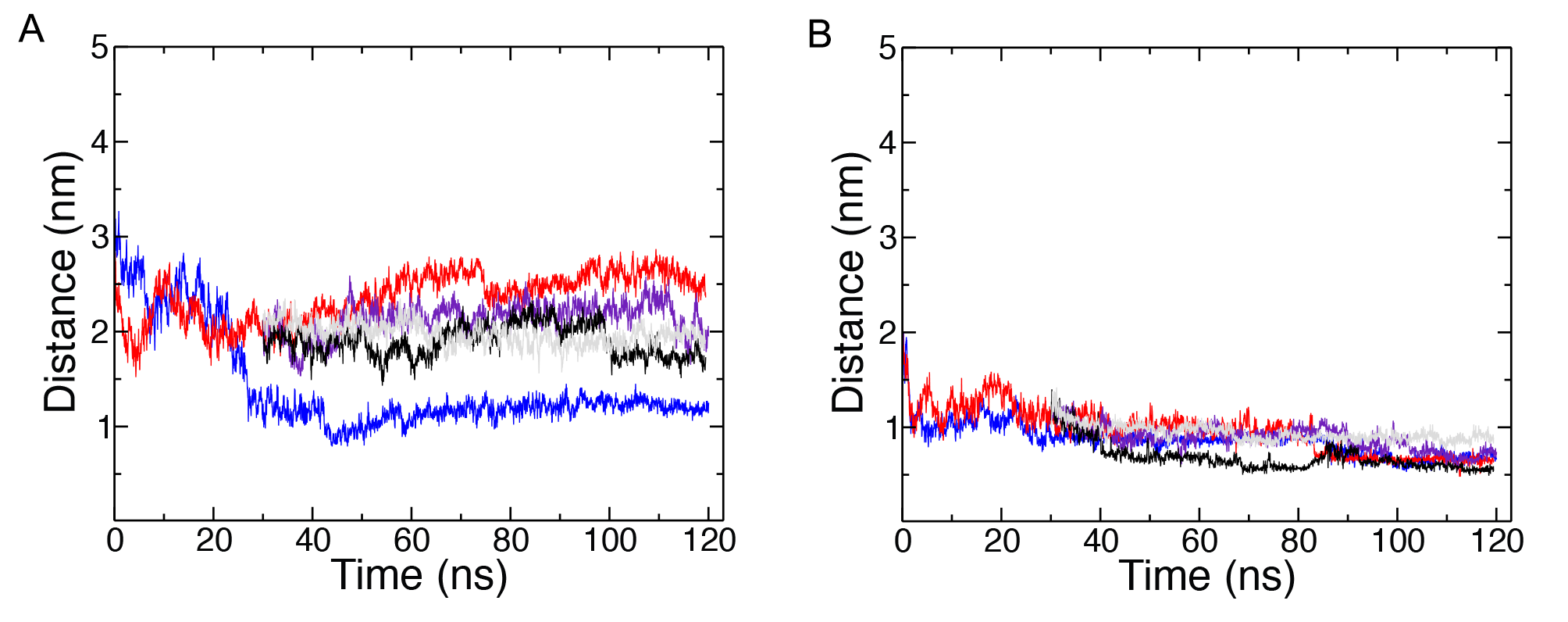

Supplement: Figure S4 — Binding of ATP to the Signature motif in the asymmetric ATP-bound simulations. The distance between the center of mass of the ATP β phosphate and that of the Signature motif serine Cα in the asymmetric ATP-bound P-gp simulations in A) ATP Binding Site 1, and B) ATP Binding Site 2 of the Run C (blue), D1 (red), D2 (purple), D3 (black) and D4 (grey) simulations. (TIF) [file pone.0091916.s004.tif]

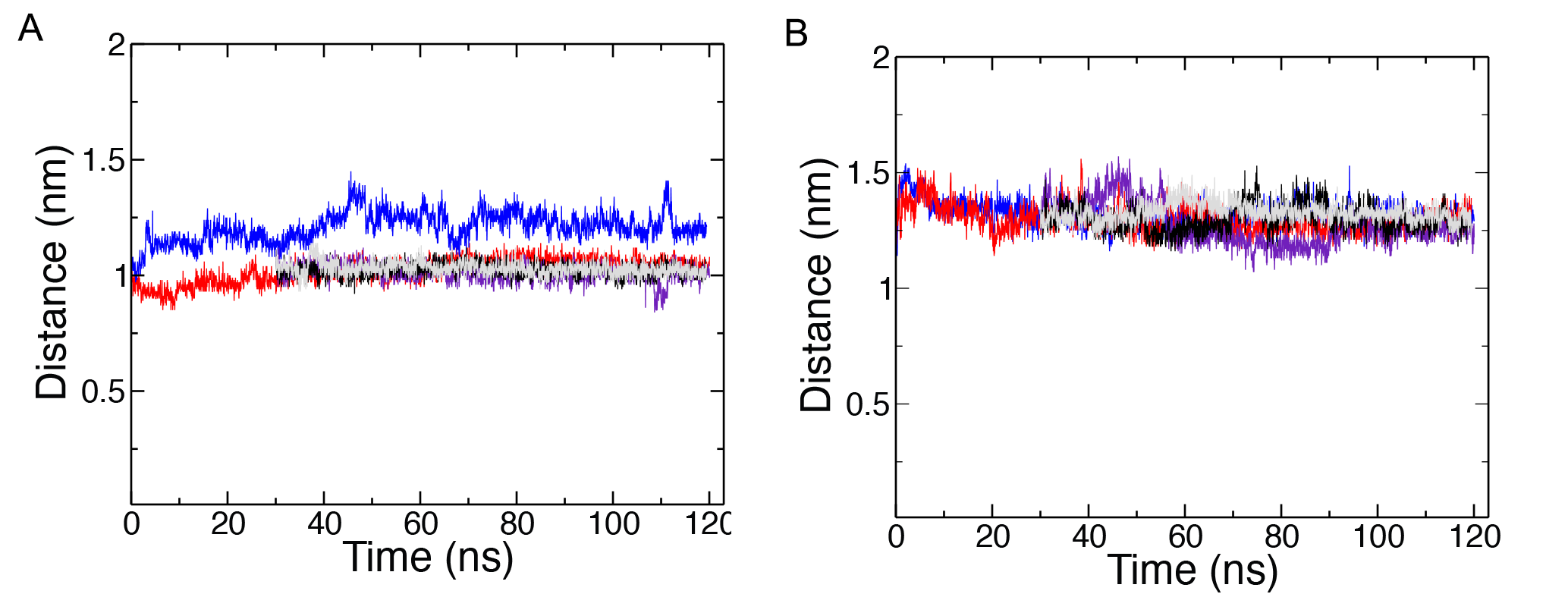

Supplement: Figure S5 — Distance between the Walker A and B motifs in the asymmetric ATP-bound P-gp simulations. The distance between the centre of mass of the Cα of the Walker B aspartate and that of the Walker A glycine of A) NBD1 (residue 426) and B) NBD2 (residue 1069) calculated throughout the MD trajectories of Run C (blue) D1 (red), D2 (purple), D3 (black) and D4 (grey). (TIF) [file pone.0091916.s005.tif]
